# Nonlinear Elasticity Assessment with Optical Coherence Elastography for High-Selectivity Differentiation of Breast Cancer Tissues

**DOI:** 10.3390/ma15093308

**Published:** 2022-05-05

**Authors:** Ekaterina V. Gubarkova, Aleksander A. Sovetsky, Lev A. Matveev, Aleksander L. Matveyev, Dmitry A. Vorontsov, Anton A. Plekhanov, Sergey S. Kuznetsov, Sergey V. Gamayunov, Alexey Y. Vorontsov, Marina A. Sirotkina, Natalia D. Gladkova, Vladimir Y. Zaitsev

**Affiliations:** 1 Institute of Experimental Oncology and Biomedical Technologies, Privolzhsky Research Medical University, 10/1 Minin and Pozharsky Sq., 603950 Nizhny Novgorod, Russia; strike_gor@mail.ru (A.A.P.); sirotkina_m@mail.ru (M.A.S.); natalia.gladkova@gmail.com (N.D.G.); 2 Institute of Applied Physics of the Russian Academy of Sciences, 46 Ulyanova St., 603950 Nizhny Novgorod, Russia; alex.sovetsky@mail.ru (A.A.S.); lionnn52rus@mail.ru (L.A.M.); matveyev@ipfran.ru (A.L.M.); vyuzai@ipfran.ru (V.Y.Z.); 3 Nizhny Novgorod Regional Oncologic Hospital, 11/1 Delovaya St., 603126 Nizhny Novgorod, Russia; dr.vorontsovdmitriy@rambler.ru (D.A.V.); zunek@mail.ru (S.S.K.); gamajnovs@mail.ru (S.V.G.); doctorvorontsov@mail.ru (A.Y.V.); 4 Department of Pathology, Privolzhsky Research Medical University, 10/1 Minin and Pozharsky Sq., 603950 Nizhny Novgorod, Russia

**Keywords:** compression optical coherence elastography (C-OCE), nonlinear elasticity, breast cancer, breast tissues

## Abstract

Soft biological tissues, breast cancer tissues in particular, often manifest pronounced nonlinear elasticity, i.e., strong dependence of their Young’s modulus on the applied stress. We showed that compression optical coherence elastography (C-OCE) is a promising tool enabling the evaluation of nonlinear properties in addition to the conventionally discussed Young’s modulus in order to improve diagnostic accuracy of elastographic examination of tumorous tissues. The aim of this study was to reveal and quantify variations in stiffness for various breast tissue components depending on the applied pressure. We discussed nonlinear elastic properties of different breast cancer samples excised from 50 patients during breast-conserving surgery. Significant differences were found among various subtypes of tumorous and nontumorous breast tissues in terms of the initial Young’s modulus (estimated for stress < 1 kPa) and the nonlinearity parameter determining the rate of stiffness increase with increasing stress. However, Young’s modulus alone or the nonlinearity parameter alone may be insufficient to differentiate some malignant breast tissue subtypes from benign. For instance, benign fibrous stroma and fibrous stroma with isolated individual cancer cells or small agglomerates of cancer cells do not yet exhibit significant difference in the Young’s modulus. Nevertheless, they can be clearly singled out by their nonlinearity parameter, which is the main novelty of the proposed OCE-based discrimination of various breast tissue subtypes. This ability of OCE is very important for finding a clean resection boundary. Overall, morphological segmentation of OCE images accounting for both linear and nonlinear elastic parameters strongly enhances the correspondence with the histological slices and radically improves the diagnostic possibilities of C-OCE for a reliable clinical outcome.

## 1. Introduction

Compressional optical coherence elastography (C-OCE) is an emerging tool used to assess elastic properties of biological tissues with a resolution of a few tens of micrometers due to a fairly high (micrometer-scale) resolution of the basic visualization method, optical coherence tomography (OCT) [1,2]. The development of C-OCE has been strongly influenced by the elastographic modality in medical ultrasounds (US), where elastography based on the compression principle was proposed in 1991 [3], and in the last two decades the US-based elastography became routinely used in clinic. This new modality radically enhanced the contrast of ultrasonic detection of tumors and improved their assessment (see, e.g., [4,5,6]).

In OCT, the elastography-related studies were triggered about a decade later than in ultrasound after a paper [7] published by Schmitt in 1998. The key point in the realization of C-OCE is the estimation of axial strains in the soft tissue, in which approximately uniaxial stress is created by compressing the studied region by the output window of the OCT probe [3]. The elastography method used in the present work is based on phase-sensitive OCT, the signal of which is highly sensitive to motions of scatterers in the tissue [8,9]. Appropriate processing of phase-sensitive OCT signals makes it possible to quantify and map local strains in the tissue [10,11,12,13].

Quantitative estimation of the tissue elasticity in compressional OCE is based on the comparison of strain in the examined tissue and the precalibrated reference layer, which is usually made of weakly scattering silicone. It is placed between the OCT probe and the compressed tissue and plays the role of optical stress sensor. Conventionally, the elasticity of biological tissue is characterized by the Young’s modulus (of shear modulus) [3,7] measured at small strains and described in the framework of the linear theory of elasticity. However, mechanical measurements of the elasticity of biological tissues (see, for example, [14]) indicated that the stress–strain dependence of these tissues can be pronouncedly nonlinear. In other words, the current elastic modulus of the tissue essentially depends on the applied stress and current strain. Recently, it was clearly demonstrated that silicones used as reference layers in C-OCE are highly linear materials in contrast to biological tissues [15,16]. Therefore, the strain of the reference silicone is linearly proportional to the applied stress [17], so that by simultaneously measuring strains in the reference silicone layer and the underlying compressed tissue, one can readily obtain and quantify the nonlinear stress–strain relationship for the tissue in a broad strain range. Examples of nonlinear stress–strain curves obtained by such a method with linear reference layers are given in [15,16,17]. Independently, similar nonlinear curves were demonstrated by combining OCT-based approximate estimates of strain with stress estimation by a force sensor [18].

Although in standard elastographic examinations based on US-platforms that enable compression elastography (also often called “strain elastography” [6]) the interpretations in terms of strain ratios ignore the tissue nonlinearity, there are some known strain-elastography-based demonstrations of pronounced nonlinearity of biological tissues [19,20]. In particular, it was shown that the spatial strain distributions in breast tissues may radically change depending on the degree of precompression of the tissue [21,22]. However, up to now, standard US-scanners based on measuring strain ratios do not enable quantitative control of precompression. The same relates to shear-wave US-elastography enabling quantitative estimates of the elastic modulus without accounting for possible tissue nonlinearity. Although both widely used variants of US-elastography (compression-based strain elastography and shear-wave realizations) have already proven their high utility in biomedical diagnostics, there are numerous examples of erroneous diagnostic conclusions that are based on estimations of the elastic modulus only. The point is that the characteristic ranges of either strain ratios or absolute values of the estimated elastic modulus may overlap for different tissue types (e.g., for benign and malignant lesions). Another closely related issue is that for the same tissue type, its elastic properties may pronouncedly (up to several times) vary depending on precompression.

In this regard, utilization of quantitative C-OCE attracted much attention in recent years for examining very heterogeneous tissues such as cancerous lesions. OCE has been investigated for imaging the biomechanical (stiffness) properties of breast [23,24,25] and other cancers *ex vivo* [26], with potential applications in tumor-margin detection [27,28]. In some works [16,17,18], possibilities of quantitative C-OCE were demonstrated for obtaining nonlinear stress–strain dependencies for biological tissues. In particular, this ability of C-OCE was used for estimation of the tissue elastic modulus under controllable pressure [17].

Experimental results, such as in [25], in which quantitative C-OCE was combined with controllable precompression, demonstrated a high potential for differentiating subtypes of breast cancer tissues using differences in their elastic behavior, even if the elastic moduli of those tissues were pronouncedly pressure-dependent because of nonlinearity. Studies in this direction are of high importance because female breast cancer has surpassed lung cancer as the most commonly diagnosed cancer [29]. It is known that typically breast cancer tumors exhibit increased stiffness, but at the same time such tumors are very mechanically heterogeneous and may strongly differ in their morphological and molecular genetic characteristics [30]. Despite the high utility of the abovementioned conventional US-based elastographic techniques, they do not utilize information about the tissue nonlinearity (and in standard examinations US scanners with elastographic modality are not able to obtain such information). In addition, the resolution of conventional US-based techniques is not sufficient to characterize the abovementioned rather fine mechanical heterogeneity of tumors. At the same time, significantly higher resolution OCE combined with the ability to differentiate grades and/or morphomolecular subtypes of tumors (which may be further improved by simultaneous accounting for the differences in their linear and nonlinear elastic properties) opens very promising prospects for solving such an important problem as the intraoperative detection of a clean boundary for tumor resection during surgical interventions.

In what follows, we describe an advanced variant of quantitative OCE enabling access to previously unavailable important diagnostic information. The main novelty in the described OCE-based diagnostics is that we combined the estimations of the Young’s modulus and the nonlinearity parameter. The latter helps to discriminate the breast tissue’s components even if the ranges of their Young’s moduli significantly overlap.

In this context, the aim of the present study was to demonstrate that the application of the developed, advanced variant of quantitative C-OCE enables quantification of tissue stiffness under controllable pressure, as well as quantitative characterization of the tissue nonlinearity. We demonstrated that the combined assessment of stiffness and elastic nonlinearity is very promising for enhancing the accuracy of differentiation of breast cancer subtypes, even if the characteristic ranges of the conventionally estimated Young’s modulus essentially overlaps for these lesions. It was also demonstrated that the combined usage of both linear stiffness values and nonlinear elastic parameters improves the reliability of tumor boundary visualization. Furthermore, in the development of the automated method of morphological segmentation of C-OCE images based on differences in the tissue stiffness [25,31], in the present study we demonstrated an advanced variant of the OCE-based automated morphological segmentation taking into account both linear and nonlinear elastic parameters.

## 2. Materials and Methods

### 2.1. OCE Imaging and Assessment of Tissue Nonlinearity

OCE data were acquired with a custom-made 20 kHz spectral-domain OCT system with a central wavelength of 1.3 µm and spectral width of 100 nm, having an axial resolution of ∼10 μm, lateral resolution of ∼15 μm, and enabling a visualization depth of 2 mm in air. A similar system was described in [32,33], where it was used to realize optical coherence angiography. OCT scans 256 × 256 pixels in size covered 4 mm in the lateral direction (with the possibility of stitching several scans in the lateral direction to cover up to 20 mm).

A variant of compressional OCE described in [15,16,17] was used to visualize local interframe strains, as well as cumulative strains produced in the tissue by compressing it with the OCT probe. The OCT probe diameter was 10 mm and the visualization depth was always an order of magnitude smaller, such that the condition of depth-independence of the compression-produced stress held quite well in the OCE measurements [15]. For estimating the axial strains required for reconstruction of the Young’s modulus in the compression approach proposed in [3], local gradients of interframe phase variations were calculated using a robust vector method developed in [11,12,34].

**Figure 1:**
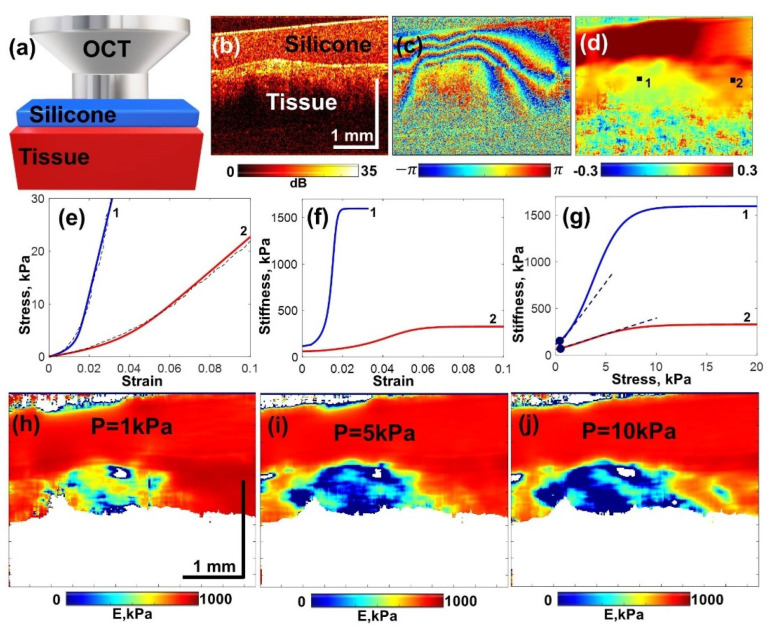
Procedures of evaluating linear and nonlinear elastic properties based on C-OCE with reference layers of highly linear silicone. (**a**) schematically shown tissue sample compressed by the OCT probe through the intermediate layer of reference silicone; (**b**) an example of a typical structural image obtained in such measurements; (**c**) an example of the derived color-coded interframe phase variation; (**d**) spatial distribution of strain obtained by estimating axial gradients of the interframe phase variation; (**e**) dashed lines show nonlinear stress–strain curves for positions corresponding to labels “1” and “2” in panel (**d**), the approximating functions are shown by the solid curves; (**f**) dependences of the tangent Young’s modulus (stiffness) on strain found by differentiation of the fitting curves from panel (**e**); (**g**) stiffness–strain dependences from panel (**f**) recalculated in stiffness–stress dependences, for which the slopes (see the dashed straight lines coming from the low-stress points ~0.5 kPa) correspond to the dimensionless nonlinearity parameter defined by Equation (4); panels (**h**–**j**) show how the reconstructed stiffness distribution evolved with increasing pressure for 1 kPa, 5 kPa, and 10 kPa, respectively.

For quantification of tissue stiffness in compressional OCE, a calibration (silicone) layer with a preliminary calibrated stiffness was used as described in detail in [15,17]. The silicone with the Young’s modulus in the range of 50–100 kPa was found to be the most suitable for studying breast tissue stiffness variations in a range from 20 kPa to ~1000 kPa or even greater. As schematically shown in Figure 1a, the reference silicone layer with a known stiffness was placed on the tissue surface and used as a sensor of the local stress. The OCT probe attached to an automated positioning stage (Purelogic R&D PLRA4, Voronezh, Russia) was slightly pressed onto the studied tissue, and the resultant strain distribution under the OCT probe was reconstructed in the same manner in both the weakly scattering reference silicone and the examined tissue under the silicone.

A typical example of the acquired structural OCT scan is shown in Figure 1b, where the silicone and underlying tissue are clearly seen. Figure 1c shows a color-coded example of interframe phase variations, the vertical gradient of which are proportional to the interframe strains. The next, Figure 1d, shows the reconstructed distribution of cumulative strains found by processing a series of several tens of OCT scans.

It has been carefully verified in previous works [15,16] that the elastic behavior of silicones is highly linear, so that incremental strains in silicone can be considered linearly proportional to the applied pressure (stress) up to fairly large cumulative strains of several tens of percentage. Therefore, the reference silicone can serve as a full-optical sensor of the local stress [17]. The inhomogeneity of cumulative strain (and, therefore, stress) in homogeneous silicone is clearly seen in Figure 1d. Because of the high nonlinearity of the majority of real biological tissues, the stress variations over the visualized region may result in unpredictable variations in the elastic modulus.

To exclude this nonlinearity-related ambiguity, we developed a procedure of pressure standardization based on analysis of several tens of OCT images of the monotonically compressed tissue and reference layer. Then interframe strains were calculated, as well as cumulative strains in both the tissue and reference layer as a function of frame number. By plotting cumulative strain in the silicone (in which it was linearly proportional to stress) against cumulative strain in the tissue we, obtained stress–stress dependences as illustrated in Figure 1e. Such dependences are usually pronouncedly nonlinear. In principle, such curves can be obtained for every point of the tissue within the OCE scan, although for reducing measurement noise and obtaining more stable results, the plotted strains were usually averaged over a fairly small processing window. For the described system, the averaging-window size was ~90–100 µm. One half of the window size defined the strain-mapping resolution ~45–50 µm. The current (also called tangent) Young’s modulus for nonlinear tissue is proportional to the slope of stress–strain curves similar to those shown in Figure 1e. Thus, by differentiating the initially measured stress–strain curve, one obtains the Young’s modulus as a function of the current strain in the tissue. In practice, the experimentally measured stress–strain curve was fitted to eliminate the influence of measurement noises, and then the approximating curve was differentiated. The result of such differentiation corresponding to Figure 1e is shown in Figure 1f. However, since the local strain in the tissue may vary even for the same lateral coordinate, it is reasonable to replot the current Young’s modulus as a function of pressure (Figure 1g) because the latter is nearly invariable at a given lateral position.

The above-described procedure of obtaining spatially resolved stress–strain dependences and the derived stiffness–stress dependences makes it possible to reconstruct spatial distribution of the Young’s modulus for a chosen value of pressure, the same over the entire visualized region. Figure 1h–j corresponding to a standardized pressure of 1 kPa, 5 kPa, and 10 kPa clearly demonstrate that because of intrinsic nonlinearity of the tissue, the apparent distribution of the Young’s modulus may drastically differ for moderate variations in the pressure. These examples qualitatively illustrate the importance of nonlinearity manifestation.

The next point was how to quantify the tissue nonlinearity to consider it as a useful informative characteristic rather than a complicating factor in the estimation of the Young’s modulus. The fact of nonlinearity is clearly seen in Figure 1e, where the stress–strain curves pronouncedly deviate from the linear dependences. In Figure 1f,g for the Young’s modulus, the nonlinearity manifests itself in the occurrence of the dependence of the tissue stiffness on strain and, consequently, on current stress. In the theory of elasticity, nonlinear stress–strain dependences are often represented as power-law expansions. In biomechanics, various more complex laws, in particular those containing exponential functions, are often used (for example, Neo-Hookean law or Verdona–Weston constitutive law and others [18,35,36]). However, different types of tissue may require different forms of constitutive law and their corresponding approximating functions. To enable a fairly universal interpretation for various form of nonlinear behavior in our approach, we adopted a local power-law approximation of the stress–strain curves around some chosen pressure value $\sigma_{0}$. Thus, retaining only the lowest-order nonlinear correction this dependence takes the form:

(1) $$\sigma =\sigma_{0}+E(\sigma_{0})\cdot (\varepsilon +\beta \varepsilon^{2}+\ldots )$$

where σ is the stress (pressure), ε is strain, $\sigma_{0}$ is the chosen initial stress around which the nonlinear stress–strain dependence is expanded, $E(\sigma_{0})$ is the current (tangent) Young’s modulus corresponding to the chosen stress $\sigma_{0}$, and β is the dimensionless nonlinear parameter characterizing quadratic-in-strain nonlinearity.

Next, the current value of the Young’s modulus by definition corresponds to the slope of the stress–strain dependence, such that at the chosen precompression one can write

(2) $$E(\sigma_{0})=d\sigma /d\varepsilon {|}_{\sigma =\sigma_{0}}$$

In other words, at any point $\sigma_{0}$, the Young’s modulus is equal to the slope of the stress–strain dependence, examples of which are shown in Figure 1e. As is clear from Equation (1), in the vicinity of point $\sigma_{0}$, the current Young’s modulus can be represented as

(3) $$E(\sigma )=E(\sigma_{0})\cdot (1+2\beta \varepsilon )$$

Next, for the quadratic nonlinearity parameter β near the stress point $\sigma_{0}$, it follows from Equations (1)–(3) that

(4) $$\beta {|}_{\sigma =\sigma_{0}}=\frac{1}{2}\frac{dE}{E(\sigma_{0})d\varepsilon }=\frac{1}{2}\frac{dE}{d\sigma }{|}_{\sigma =\sigma_{0}}$$

In other words, Equation (4) indicates that the nonlinearity parameter $\beta {|}_{\sigma =\sigma_{0}}$ is equal to 1/2 of the slope of dependence E(σ) at point $\sigma_{0}$ (examples of such slopes for the experimentally obtained curves E(σ) are shown in Figure 1g by dashed lines). It can be noted that such expansion can be made around arbitrarily chosen initial stress, in particular zero pressure $\sigma_{0}\to 0$, may also be chosen as the initial point.

**Figure 2:**
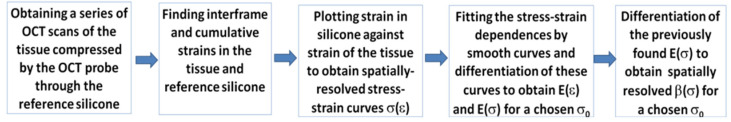
Schematic of the main steps of signal processing to estimate stiffness and the nonlinearity parameter in the developed C-OCE.

The steps required for obtaining spatial distributions of the Young’s modulus $E(\sigma_{0})$ (stiffness) and nonlinearity parameter $\beta {|}_{\sigma =\sigma_{0}}$ in the described variant of C-OCE can be summarized as schematically shown in the flowchart in Figure 2.

### 2.2. Principle of Segmentation of OCE-Images Using Linear and Nonlinear Elastic Parameters

We applied the procedure of automated morphological segmentation of the OCE images using the preliminary determined ranges of both the linear Young’s modulus and nonlinear elastic parameter for different morphological components of the breast tissue.

Our previous studies showed that, for a chosen “standardized” stress, various morphological components of tumorous tissues and peritumoral zones often exhibit quite well-separated characteristic stiffness ranges [25,31,37]. These characteristic stiffness ranges can be found by comparing OCE-based stiffness maps and conventional histological images obtained for the same locations. After determining the characteristic stiffness range for a certain morphological component of the tissue, we considered that every morphological component corresponds to those pixels in the OCE image, for which the stiffness falls in the respective characteristic stiffness range. In such a way, the areas corresponding to every component can be automatically segmented and marked by various colors in the OCE-based stiffness map. In study [31], such quite well-separated stiffness ranges were found for four mains constituents of model tumors (namely, regions of viable cancer cells, necrotic cancer cells, benign fibrosis or adipose in peritumoral zones, etc.). The results of segmentation of OCE maps of the Young’s modulus according to those four characteristic stiffness ranges demonstrated a very good agreement with manually performed segmentation of histological images. Similar segmentation of OCE images based only on the characteristic ranges of the Young’s modulus was efficiently applied in [37] to another type of model animal tumors, for which the same four main morphological components were also typical.

However, in OCE studies of breast cancer samples of patients such as in [25,38] had some cases where histological examinations indicated the presence of morphologically different zones, for which the characteristic ranges of the Young’s modulus demonstrated a significant overlap. In such situations, however, one may expect that even if these tissue components are fairly similar in terms of their Young’s moduli, the nonlinear parameters for these components may differ, which can be additionally used for the differentiation. There is a general analogy of this idea with nonlinear-acoustic diagnostics of structural damage in mechanical engineering, where cracks and cavities may have similar scattering strength, but cracks may still be clearly differentiated because pronounced nonlinearity-generated sounding-signal components are specific only for cracks [39].

Therefore, in addition to consideration of only Young’s modulus maps, similar maps can be constructed for the nonlinearity parameter that can be determined as described in the previous section. Then the segmentation of the morphological components on OCE maps can be made much more reliably by simultaneously using their differences in both the Young’s modulus and the nonlinearity parameter. In the “Results” section, we will demonstrate the realization of this idea for improved diagnostics of breast-cancer samples.

### 2.3. Patient Selection and Data Collection

The present study on human tissue samples was approved by the Institutional Review Board of the Privolzhsky Research Medical University (Nizhny Novgorod, Russia). All of the patients included in the study provided written informed consent. The research was carried out on 60 specimens of freshly excised breast tissue acquired from 50 patients post breast-conserving surgery with different histopathology diagnosis (see Section 2.4). During resection, tumorous tissue specimens were taken from different parts of the tumor—in the center and peritumoral (nontumoral) area. All specimens were studied within 1–2 h after resection.

### 2.4. Histopathology and Its Comparison with OCE-Based Segmentation

After C-OCE imaging, the scanning area was marked on the specimen with histological ink for easier correlation with histology. Then, the specimens were fixed in 10% formalin for 24 h and were transferred to 70% ethanol and then paraffined for histological study. The paraffined specimen blocks were sliced through the marked area to match the plane of histological sections with the OCT B-scan position.

To determine the disease type and collagen content, staining of the histological slides with hematoxylin and eosin (H&E) and Van-Gieson’s was performed. The histological slices were prepared using a Leica RM 2245 Rotary Microtome, described by a morphologist and photographed in transmitted light with a Leica DM2500 DFC (Leica Microsystems, Germany) microscope, equipped with a digital camera.

The revealed histological types of breast tissue included adipose tissue with streaks of connective tissue (number of specimens n = 7); fibrous stroma (n = 9); area of invasive ductal carcinoma (IDC), which is characterized by individual tumor cells and dense fibrous stroma with hyalinosis of collagen fibers (n = 10); area of IDC, consisting of separate large clusters or nests of tumor cells distributed in the fibrous stroma (n = 11); agglomerates of tumor cells of IDC, which is characterized by solid growth with a small amount of stroma (n = 11); agglomerates of tumor cells of invasive lobular carcinoma (ILC), which is characterized by solid growth (n = 6); and large foci of hyalinosis (n = 6). The results of the histopathology were compared with the OCE findings.

When performing such comparison/matching, one should bear in mind that neighboring OCE B-scan and histological slices may have variations in the visualized morphological tissue structure on a scale of a few tens of microns. Such small-scale features could not be colocated in OCE scans and histology because even for accurate labeling of the scanned zone with histological inks, colocation of OCE scans and histological images could be made with an accuracy of a few hundred microns at best. Furthermore, the applied compression deformed the sample during the OCE examination, and then the shape of the sample was additionally distorted during the procedures of its preparation for the histological examinations (fixation in formalin, dehydration, paraffining, etc.). In view of this, in this study, in order to obtain a good correlation between OCE-scans and histological sections, rather large regions of interest (~hundreds of microns or more) with fairly uniform histological properties were used for comparison and precalibration of the linear and nonlinear elastic parameters. Such larger regions did not exhibit significant variations from one histological slice to another, so that precalibration of the characteristic ranges of the Young’s modulus and the nonlinearity parameter was possible. Then the regions corresponding to the found characteristic ranges of the two parameters were easily automatically singled out on the initial continuous maps of the Young’s and nonlinear parameter.

Therefore, for samples with more a complex structure, the correspondence of OCE-segmentation and histology mainly concerns similar zones’ topology and only an approximate similarity of geometrical forms, especially for smaller details in the images.

## 3. Results

**Figure 3:**
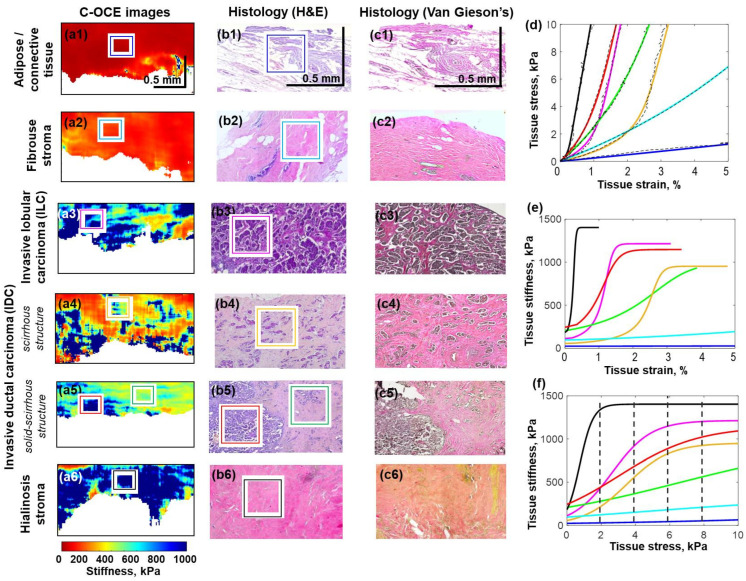
Illustration of different nonlinear elastic behaviors for seven different types of benign and malignant breast tissues, for which the representative zones are marked by different colored rectangles on histological and OCE images. Column (**a1**–**a6**) shows C-OCE images obtained for a standardized pressure of 4 kPa. Columns (**b1**–**b6**, **c1**–**c6**) show histological H&E-stained and Van-Gieson-stained images, respectively. Typical stress–strain curves for the seven tissue zones marked by different colored rectangles are shown the same color curves in panel (**d**). (**e**) shows the corresponding derived stiffness–strain curves and (**f**) is for the stiffness–stress curves. Notice that malignant tissues (red, pink, orange, and green lines) tend to exhibit larger nonlinearity than benign tissues (blue and light-blue lines). Blue lines—adipose tissue with layers of connective tissue; light blue lines—fibrous stroma; red lines –agglomerates of tumor cells of IDC; orange lines—area of IDC, consisting of separate large clusters or nests of tumor cells distributed in the fibrous stroma; green lines—area of IDC, which is characterized by scattered individual tumor cells and dense fibrous stroma with hyalinosis of collagen fibers; pink lines—agglomerates of tumor cells of ILC, which is characterized by solid growth; black lines—large foci of hyalinosis. (Abbreviations: IDC—invasive ductal carcinoma; ILC—invasive lobular carcinoma).

*Ex vivo* OCE imaging of excised breast cancer samples was performed using a motorized stage enabling the positioning of the OCT probe in the lateral plane and along the vertical axis to apply compression to the samples. The technique based on the application of reference silicone layers described in Section 2.1 made it possible to obtain stress–strain, stiffness–strain, and stiffness–stress curves for every type of the studied tissues, as shown in Figure 3. It was shown that there is a significant difference in the stiffness and the rate of increase in stiffness depending on strain (and stress) among nontumorous and various types of tumorous breast tissues. The latter were characterized by significantly differing rates of malignancy and the conventional way of differentiation of these cancer subtypes is based on laborious and time-consuming histological examination. In view of this, revealing clear differences using OCT-based examinations of freshly-excised samples has a high interest.

Figure 3d–f show the results for six types of breast tissues demonstrating significantly different types of corresponding stress–strain dependences. It is clear that only nontumorous adipose (row 1 in Figure 3) exhibits very pronouncedly reduced Young’s modulus and weak nonlinearity of the stress–strain dependence. The four tissue types demonstrate much higher stiffness and higher nonlinearity (of which three types in rows 2, 4, 5 in Figure 3 are malignant lesions and hyalinosis shown in row 6). The elastic properties of fibrosis (row 1 in Figure 3) are intermediate between these two strongly differing types. The characteristic forms of stress–strains curves and the derived stiffness–strain and stiffness–stress curves are shown in column 4 of Figure 3 by different colors.

It was observed that fairly large agglomerates of tumor cells (see images in the 3rd and 5th rows and red and pink curves in the Figure 3d–f), as well as large foci of hyalinosis (images in the 6th row in Figure 3 and black curves in the Figure 3d–f) are characterized by pronouncedly increased nonlinearity and stiffness in comparison with adipose (blue lines in Figure 3d–f). The light blue lines in Figure 3d–f) for the fibrous tissue clearly demonstrate the abovementioned intermediate behavior. Invasive ductal and lobular cancer is more than 20 times as stiff as normal adipose tissue at 0.1% strain and more than 200 times as stiff at 2% strain. Compared to normal fibrous tissue, those types of cancer are more than 10 times as stiff at 0.1% strain and nearly 100 times as stiff at 2% strain. Therefore, relative stiffness is a fairly good parameter for differentiating such tissue types, although not always. Indeed, the stiffness–strain and stiffness–stress curves in Figure 3e,f demonstrate that either at the lowest pressures or some intermediate pressure levels the Young’s modulus may coincide for different cancer types.

Next, the targeted comparison of OCE images and histological ones shown in rows 4 and 5 in Figure 3 revealed regions in which small islands of cancer cells were embedded in fibrous stroma and dense fibrous stroma with hyalinosis and collagen fiber. These structures correspond to tumors with scirrhous and solid-scirrhous structure types. It was found that such regions are characterized by an intermediate nonlinearity and stiffness (green and orange lines in the Figure 3d–f) between regions with large agglomerates of cancer cells (“solid tumors”) and hyalinosis on the one hand and fibrous stroma and adipose on the other hand. It should be mentioned that scirrhous tumors with inclusions of individual cancer cells into fibrous stroma at the lower pressures may exhibit fairly low stiffness similar to that of nontumorous fibrous tissue, so that based on the similarity of stiffness values these two tissue types can be easily confused.

However, despite the initially similar stiffness at low pressures, the dependence of the Young’s modulus on pressure is noticeably different for these tissue types, which can help to distinguish fibrosis from the tissue containing only sparsely distributed cancer cells visible in the histological images (Figure 3(b4,b5)). This is important for precise diagnostics of such scirrhous tumors, as well as for searching out a clear resection margin during organ-conserving surgical excision of breast cancer.

In addition, the described method of OCE examination made it possible to clearly single out regions of stroma with pronounced hyalinosis (the presence of thick bundles of collagenous fibers corresponding to the histological images in row 6 of Figure 3). Occurrence of hyalinosis in stroma indicates the development of deep secondary degenerative alterations in the tissue, which is important for diagnosing the disease and its stage, as well as for planning the therapy. Our study revealed that hyalinosis is characterized by high stiffness values (similar to those for solid agglomerates of cancer cells), but the nonlinearity of hyalinosis is much higher (black line in Figure 3f). This extremely high nonlinearity can be attributed to rather high packing density of the collagen fibers resembling the hyaline cartilage, such that under very small strains (~0.1%) the residual gaps become closed and the stiffness of such hyalinosis zones drastically increases approaching the stiffness of cartilage.

Therefore, the performed OCE-based study revealed that there is a significant difference both in the stiffness and in the rate of stiffness increase with strain among the examined tumorous and nontumorous breast tissues.

**Figure 4:**
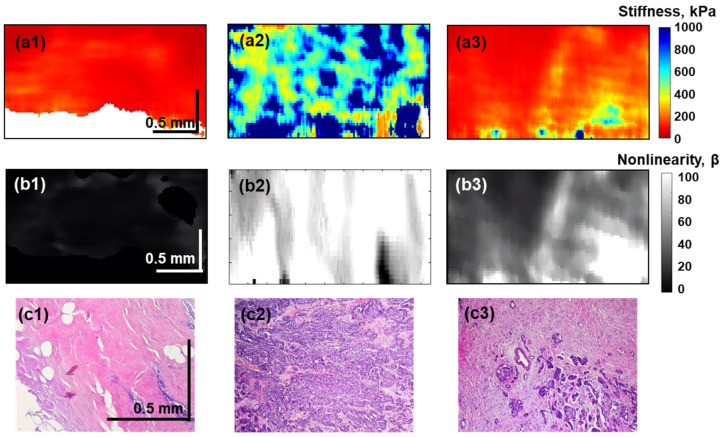
OCE-based visualization of the Young’s modulus *E*_0_ (row (**a**)) and the nonlinearity parameter *β* (row (**b**)) obtained for low pressure 0.5 ± 0.5 kPa applied to the tissues, for which the corresponding histological images are shown in row (**c**). (**a1**–**c1**) is a benign fibroadenoma, (**a2**–**c2**) is a malignant tumor of solid type and (**a3**–**c3**) is a malignant tumor of scirrhous type. Notice that benign fibrosis and scirrhous tumor exhibit very similar stiffness (compare panels (**a1**,**a3**)), but distinctly different nonlinearity parameters (panels (**b1**,**b3**)).

In another form, the difference in the diagnostic information given by the Young’s modulus and the nonlinearity parameter is demonstrated in Figure 4 for benign and malignant breast lesions: benign fibrosis, solid-type malignant tumor, and scirrhous cancer, for which the histological images are shown in row 3. Rows 1 and 2 in Figure 4 show the color-coded spatial distributions of the Young’s modulus and the grey-level coded dimensionless nonlinearity parameters, respectively. These elastic parameters were calculated according to Equations (2) and (4) for the low applied pressures (about 0.5 ± 0.5 kPa). Figure 4 shows that for the solid-type tumor, both the Young’s modulus and the nonlinearity parameter are strongly elevated, whereas for fibrosis and the scirrhous tumor, the Young’s modulus demonstrates very similar fairly low values. However, Figure 4 also clearly shows that for the scirrhous tumor, the nonlinearity parameter (albeit smaller than for the solid tumor) is pronouncedly higher than for fibrosis. These examples clearly illustrate the statement that the estimation of the linear elastic modulus (i.e., its value conventionally estimated at fairly low pressures typical of conventional ultrasound elastography) may be insufficient for discrimination between malignant and benign breast lesions; whereas due to the additional estimation of the nonlinearity parameter, accurate discrimination becomes possible.

**Figure 5:**
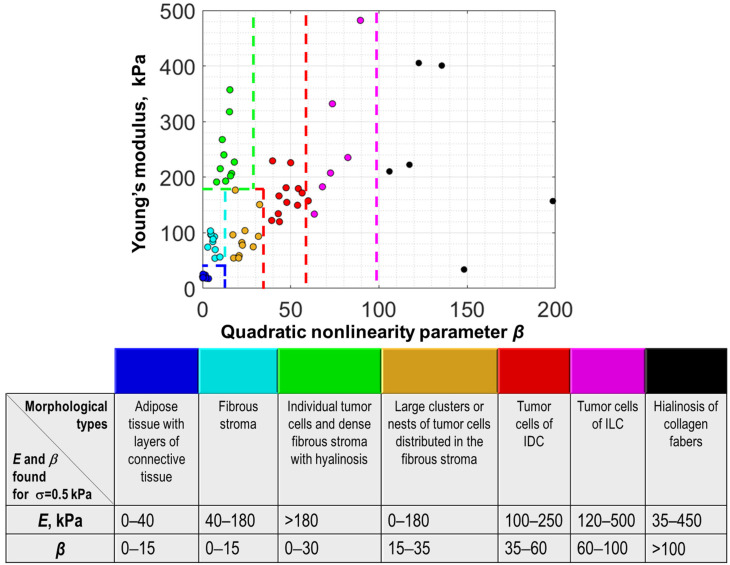
Two-dimensional plane (*E*,*β*) of the Young’s modulus and nonlinearity parameter for the same seven histologically different tissue types as in Figure 3 obtained using 60 samples taken from 50 patients. Notice that malignant tissues (green, orange, red, and magenta points) tend to exhibit stronger nonlinearity than benign tissues (blue and light-blue points); the strongest nonlinearity is typical of hyalinosis. Although the Young’s modulus of adipose is clearly the lowest, for the other tissue types, the dependence of the Young’s modulus on the malignancy degree is not that clear and strong overlap of stiffness ranges may occur. (Abbreviations: IDC—invasive ductal carcinoma; ILC—invasive lobular carcinoma).

Furthermore, the total set of results for OCE examination of 60 samples corresponding to seven different tissue types revealed in the histological images is represented in Figure 5 in the 2D form on the (*E*,*β*) plane, where the horizontal axis is for the nonlinearity parameter *β* and the vertical one is for the Young’s modulus *E.* Both quantities were estimated at lower pressures (about 0.5 ± 0.5 kPa), which was controlled using the reference silicone. The color correspondence to the tissue types is indicated in the legend. Figure 5 shows that on the 2D plane all these seven groups can be fairly well-separated (the dashed lines in Figure 5 show the corresponding boundaries of the characteristic ranges of parameters *E* and *β*). In contrast, in terms of *E* and *β* taken separately, there is a significant overlap in these parameters for several tissue types (the semitransparent ellipses in Figure 5 show some of such data with overlapping values of *E* or *β*).

**Figure 6:**
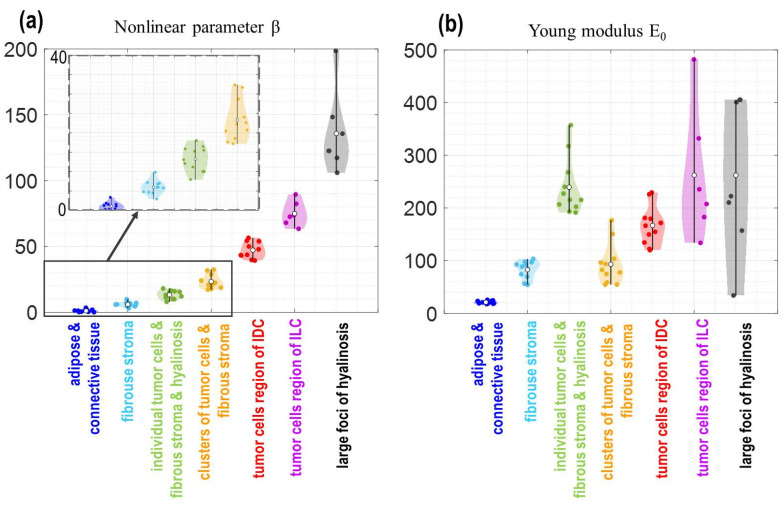
Nonlinear parameter *β* (**a**) and Young’s modulus *E*_0_ (**b**) for different types of breast tissues: blue dots—nontumor breast tissue (adipose tissue with layers of connective tissue); light-blue dots—benign fibroadenomatosis or fibroadenoma; green dots—area of IDC, which is characterized by individual tumor cells and dense fibrous stroma with hyalinosis of collagen fibers (scirrhous structure); orange dots—area of IDC, consisting of separate large clusters or nests of tumor cells distributed in the fibrous stroma (solid-scirrhous structure); red dots—area of IDC which is characterized by solid growth with a small amount of stroma; magenta dots—area of ILC, which is characterized by solid growth; black dots—large foci of hyalinosis. The inset in (**a**) shows the data for the first four tissue types in more detail. (Abbreviations: IDC—invasive ductal carcinoma; ILC—invasive lobular carcinoma).

To more clearly show the abovementioned trend of the nonlinearity increase for tumors with increasing malignancy up to the maximal values typical of hyalinosis and nonmonotonic behavior of the Young’s modulus in Figure 6, the estimated values of *E* and *β* are shown separately for the same tissue types as in Figure 5. The empty white circles in each of the data clouds show the mean values and the vertical bars correspond to show the total scattering of the data within each cloud. It is clear that when only Young’s modulus or only nonlinearity parameter is considered, there may be a significant overlap in their values for the considered tissue subtypes. Nevertheless, on the two-dimensional (*E,β*) plane these tissue subtypes can be fairly well-separated in terms of the specific ranges of *E* and *β* indicated in the legend in Figure 5.

When considering the data presented in Figure 5, it should be emphasized that both quantities *E* and *β* shown in the figure are estimated for lower pressures (in our case about 0.5 ± 0.5 kPa) and the statements about the monotonic ordering of the estimated nonlinearity parameters and nonmonotonic ordering for the Young’s modulus are made for these low-pressure results. It is clear from Figure 3 that for larger pressures, the nonlinear stress–strain curves may cross each other, for example, the red and magenta color stiffness–pressure curves shown in Figure 3f for IDC and ILC, respectively. This crossing means that one tissue type can be stiffer than the other at lower pressure, but the situation may change to the opposite at a higher pressure and vice versa. For this reason, we emphasize once again that for correct interpretation of relative stiffness values, as well as characteristic absolute values, it is critically important to enable local pressure control when the stiffness is estimated. 

The next step after determining the characteristic ranges of the Young’s modulus and the nonlinear elastic parameter based on a targeted comparison of OCE data and histological images is the application of the found characteristic values for automated morphological segmentation of the OCE images. The utilization of two parameters (Young’s modulus and nonlinearity parameter) for such segmentation is a further development of the automated morphological segmentation of OCE images that was first proposed in [25,31,37] using only the differences in the characteristic ranges of the Young’s modulus for various tissue subtypes. The combined utilization of both Young’s modulus and the nonlinearity parameter opens the possibility to distinguish the tissue types even if their characteristic stiffness significantly overlaps (as was demonstrated by comparing the 1st and 3rd columns in Figure 4). The additional information provided by the nonlinearity parameters opens the way for a more precise assessment of the breast cancer morphology. We demonstrated that it becomes possible not only to reliably delineate peritumoral adipose tissue, but also to automatically differentiate such important tissue subtypes as fibrous stroma and fibrous stroma with individual cancer cells, malignant areas of IDC and ILC, as well as zones of hyalinosis. This is made without any special preparation of freshly excised breast-tissue tissue samples.

**Figure 7:**
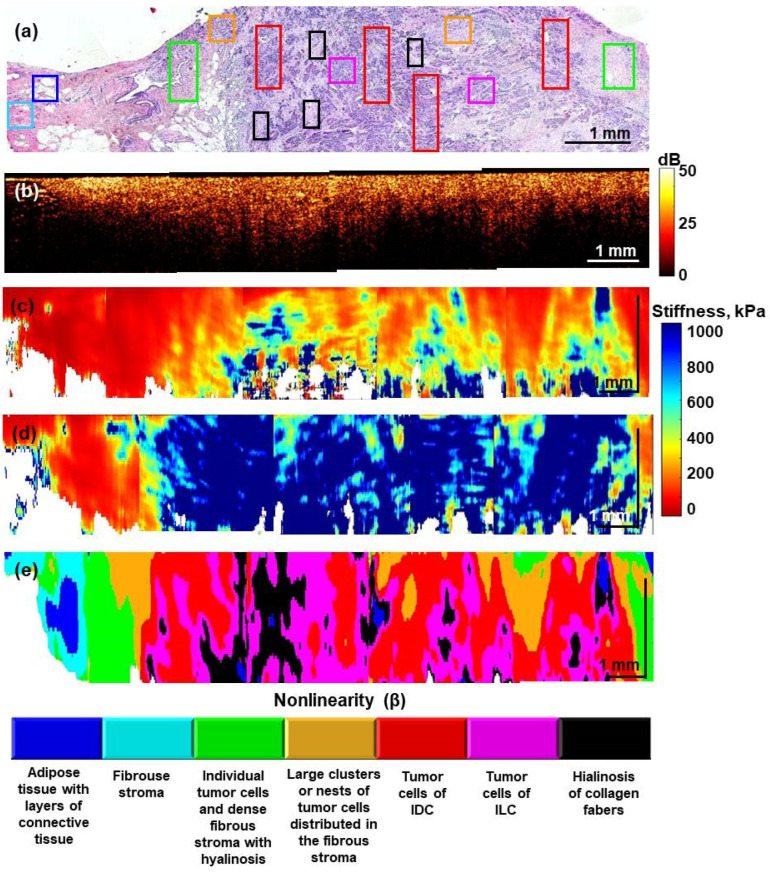
Visualization of a transitional zone between peritumoral (nontumoral) breast tissue and tumor region using structural OCT and C-OCE-based images with morphological segmentation of the OCE image. The segmentation is based on the combined usage of the Young’s modulus (*E*) and the nonlinearity parameter (*β*), for which the characteristic ranges are shown of (*E*,*β*) plane in Figure 5. (**a**) is the H&E-stained histological image; (**b**) is the structural OCT image which is obtained by stitching several scanned sections; (**c**) is the stiffness map obtained at a minimum pressure of 0.5 ± 0.5 kPa; (**d**) is the stiffness map obtained at a pressure of 4.0 ± 0.5 kPa demonstrating strong pressure-induced change in tumor stiffness; (**e**) is the automated morphological segmentation of the OCE image into zones of various tissue components. The corresponding regions in the histological image (**a**) are marked by rectangles of the same color as the segmented zones in panel (**e**). (Abbreviations: IDC—invasive ductal carcinoma; ILC—invasive lobular carcinoma).

Such an example is given in Figure 7, which shows the histological image (Figure 7a), the structural OCT image (Figure 7b), the OCE-based stiffness map obtained at two different pressures of 0.5 ± 0.5 kPa and 4.0 ± 0.5 kPa (Figure 7c,d), and finally, the morphological segmentation map (Figure 7e). The latter was constructed using the characteristic ranges of both the Young’s modulus and the nonlinear elastic parameter. These characteristic ranges were obtained by targeted comparison of the histological images and OCE data using over 60 samples that were summarized in Figure 5. The OCE and histological images in Figure 7 were obtained by stitching several sequentially scanned sections, in which the boundary between normal (peritumoral) tissue (left part of the images) and an invasive tumor (nonluminal subtype) is clearly seen.

In the conventional structural OCT images in Figure 7b, there are no clear differences between tumorous and nontumorous zones. In the OCE-based stiffness map plotted for the lowest pressures of 0.5 ± 0.5 kPa (Figure 7c), the central tumor zone becomes visible as a stiffer region, but there is still no clearly seen transition between the tumor and peritumoral zone.

The stiffness map in Figure 7d is obtained at a higher precompression (4.0 ± 0.5 kPa) and clearly demonstrates the effect on nonlinearity-induced stiffening due to which the tumor zone grows significantly stiffer. Thus, in Figure 7d, one can see a much clearer contrast between the tumor in the central part and the peritumoral adipose and stroma because the nonlinearity of the latter is much lower and causes only insignificant stiffening. Unlike Figure 7c,d, in which the quantification of nonlinearity was not yet used, the next Figure 7e shows the results of morphological segmentation of the OCE-image based on the quantitative differences in both Young’s modulus and the nonlinearity parameter (these differences for seven tissue subtypes were shown in Figure 5). In Figure 7e, the OCE-based segmentation demonstrates much clearer correspondence with the histological slide for the structure of the transition between the tumor and peritumoral zone. Now, in addition to the large agglomerates of cancer cells with differentiation of IDC and ILC (red and magenta colors, respectively) in the central tumor zone in Figure 7e and soft adipose (dark-blue) at the periphery, even finer differentiation becomes possible. Namely, inclusions of hyalinosis (black color) in the central part of the tumor and in the transition to the peritumoral zone, finer tissue subtypes are segmented: fibrosis without cancer cells (light-blue), fibrosis with scattered individual cancer cells (green), and fibrosis with embedded small clusters of cancer cells (orange). Such a visualization with a high-sensitivity detection of even sparse amounts of cancer cells is very important for controlling a clean resection boundary and becomes possible due to the combined utilization of both the nonlinearity parameter and Young’s modulus of the tissue.

However, for compared C-OCE-based images and histological images, the uncertainty of matching evidently may be significantly greater than the distance between neighboring histological slices (see Section 2.4). This important remark should be taken into account when comparing OCE and histological images: only larger (~hundreds of microns and greater) regions/components can be reasonably matched and compared on this scale, whereas smaller features (~tens of microns) may look significantly more distorted/displaced than for neighboring histological slices. Therefore, the correspondence mainly concerns similar topology and only approximate similarity of geometrical forms, especially for smaller details in the images.

Thus, the rectangles in Figure 7a show only representative regions for various tissue subtypes and the color of these rectangles corresponds to the palette used in Figure 7e to denote the segmented tissue constituents of the seven types. The similarity in topology and geometry of the corresponding zones in the histological slice is quite clear. The automated OCE surprisingly revealed the same seven morphological components as the manual segmentation of histological images performed by an experienced histopathologist.

## 4. Discussion

The majority of elastography-related studies (both in OCT and ultrasound) till now were focused on linear elastic properties (Young’s modulus) [6,8,23,25], so that interpretation of elastographic data was conventionally performed within the paradigm of the linear elasticity theory both in ultrasonic techniques widely used in clinic and in emerging OCT-based elastographic methods. However, there is an increasing number of demonstrations based on various techniques (mechanical indentation, ultrasound, OCE) which clearly indicate the pronounced nonlinearity of biological tissues (e.g., [13,15,17,18]). This nonlinearity can confound interpretation of elastographic images in both ultrasound elastography and OCE [40]. Thus, for meaningful interpretation and comparison of estimated stiffness values (for both various scan regions in one measurement and, furthermore, different measurements), one requires knowledge of pressure (stress), from which these estimates are obtained. We emphasize that even within a single scan it is necessary to know local values of the applied pressure for various lateral coordinates rather than merely an averaged stress over the entire scan, measurements of which are conventionally made by applying controllable force to the compressing probe. Thus, a key feature of the OCE method used here is that for estimating the tissue stiffness, the reference silicone layer acts as a spatially-resolved sensor, enabling knowledge of the local stress. Due to this, we were able to estimate the tissue stiffness for the same prechosen standardized range of the applied pressure, even if the instantaneous pressure distribution could unpredictably vary within a single scan (see details in [17]).

It can be also noted that in our quasistatic compression OCE and compression ul-trasound elastography, the tissue is deformed fairly slowly (corresponding to frequencies of ~several Hz in the spectral domain). A similar rate of compressive strains is used in stiffness estimations based on the utilization of loading mechanical devices, the results of which often being accepted as ground truth when validating various elastographic techniques. In this context, a recent paper [41] demonstrated a good agreement between the results of wave-based OCE using waves in ~kHz frequency range and mechanical measurements that are quasistatic in the same sense as the compression OCE technique discussed here.

Furthermore, in several previous works related to compression OCE, we demonstrated that the realization of pressure standardization in different experiments and within the visualized region in each experiment can eliminate the nonlinearity-related ambiguity in the interpretation of the estimated Young’s modulus [17,25,31,37].

In the present study, we took the next step and demonstrated that, alongside the Young’s modulus estimation under standardized loading conditions, quantification of the local nonlinear response of breast tissue can additionally provide valuable diagnostic information and enable unprecedented selectivity in elastographic differentiation of tissue subtypes.

First, quantification of the nonlinear elastic parameter in combination with the Young’s modulus improves the reliability of discrimination between benign and malignant breast tissues. The point is that benign and malignant tissues may have significantly overlapping ranges of the Young’s modulus. For example, it is demonstrated in Figure 6 that strongly overlapping ranges of the Young’s modulus are typical for yet benign fibrosis (light blue dots in Figure 6), as well as for malignant areas of IDC, consisting of separate, fairly large clusters or nests of tumor cells distributed in the fibrous stroma, i.e., tumor with solid-scirrhous structure (orange dots in Figure 6). However, these tissue types demonstrate pronouncedly differing nonlinearity parameters and thus can be quite distinctly separated on the two-dimensional (*E*,*β*) plane as shown in Figure 5.

Second, the estimates of stiffness combined with the quantification of the nonlinear elastic parameter enabled the possibility to distinguish even zones with sparsely scattered tumor cells scattered in the fibrous tissue (green dots in Figure 5 and Figure 6). We emphasize that such zones do not demonstrate clearly different stiffness from hyalinosis (black dots in Figure 5 and Figure 6) and from two other types of tumorous tissue (red and magenta dots in Figure 5 and Figure 6). To the best of our knowledge, previous detection of such tissue subtypes with sparsely distributed cancer cells was possible only using high-resolution histological images in which individual tumor cells are visible. However, the resolution of OCE is lower and OCE images do not literally resolve individual tumor cells. Nevertheless, OCE reveals that the presence of such subresolution individual cancer cells causes changes in the linear and nonlinear elastic properties of the tissue on larger scales of tens and hundreds of micrometers. In materials science, a very similar situation is known in nonlinear vibro-acoustic detection of microcracks in solids (e.g., metals or rocks). Such individual microcracks are not resolved, but the crack-induced changes in the material’s elastic nonlinearity can be easily observed, for example, via nonlinear vibro-acoustic modulation effects [39].

Returning to Figure 5 and Figure 6 and the example of OCE-segmentation in Figure 7, it is interesting to point out the possibility to clearly highlight areas of the hyalinized tumor stroma. This is possible due to the extremely high nonlinearity of hyalinosis, although its Young’s modulus strongly overlaps with that typical of zones containing large agglomerates of cancer cells.

Another condition that is of key importance for the correct interpretation of OCE results is that, for fairly soft biological tissues, quantitative estimations of both the tangent Young’s modulus *E* and the quadratic nonlinearity parameter *β* should be performed for a chosen (“standardized”) pressure. For unambiguous and reliable interpretation, this pressure should be the same not only for various compared OCE images from different experiments, but also should be maintained the same within the visualized region in every particular experiment. To ensure this requirement, conventional methods based on force sensors that enable only spatially averaged estimates of pressure are not sufficient. The reason is that, physically, the stress is not uniform even within a single OCT scan because of the influence of uncontrollable fine geometrical factors and intrinsic mechanical inhomogeneity of the tissue. For this reason, one has to acquire a series of OCT scans of the deformed tissue and then choose scans with different numbers, for which the desired stress level is attained in every region of interest of the entire visualized region. In the above-described study, this requirement was satisfied using local control of stress by measuring local strains in reference silicone layers that act as a distributed optical pressure sensor. We restate that the results presented above were obtained for fairly low pressure 0.5 ± 0.5 kPa, although other “standardized” pressure values can also be used, for example, pressure ~4 kPa was used in [31,37] and was quite appropriate for performing OCE-based segmentation of model tumors in animal experiments using only Young’s modulus. Evidently, for different tissue types, different pressure levels may be more or less efficient for obtaining better accuracy of segmentation. In this direction, further studies are required.

The results of determining the characteristic ranges of the Young’s modulus and the nonlinearity parameter in the present OCE study indicated that the relationship of the tissue malignancy and its low-pressure stiffness is neither straightforward, nor monotonic. This is clearly seen from Figure 6b showing the Young’s modulus for various tissue types including several tumors with various malignancy grades. The magnitude of the nonlinearity parameter *β* in Figure 6a appears more regularly varying, but may also demonstrate noticeable overlap, so that clearer separation requires simultaneous utilization of stiffness and nonlinearity as is clear from the two-dimensional Figure 5, in which fibrosis and IDC tumors with individual cancer cells or with larger clusters of cancer cells can be rather clearly delineated on the (*E*,*β*) plane, but may noticeably overlap along each of the two axes.

Concerning the prognosis of the diagnostic accuracy of such OCE-based differentiation, it should be said that the considered set of 60 samples is still insufficiently large for a reliable conclusion. However, for the discussed set of 60 samples, the OCE-based differentiation of all seven types of tissues shown in Figure 5 and Figure 6 gave 100% correct results.

Surely, the additional information about the tissue nonlinearity should further improve the accuracy of OCE-based diagnostics, although obtaining accurate statistically significant conclusions for discrimination of seven tissue types at once may require significant additional time for the accumulation of sufficient numbers of samples of each type and their detailed comparative OCE-based and histological examinations and analysis. In the present, actually pilot-type, study, our main goal was to demonstrate that the combined usage of linear and nonlinear elastic parameters of tissues enables unprecedented selectivity of breast tissue differentiation due to which it becomes possible to clearly discriminate seven subtypes of breast tissues. Conventional OCT imaging and even OCE based on the Young’s modulus only were not able perform such fine differentiation.

Overall, it can be already stated that morphological segmentation of OCE images accounting for both linear and nonlinear elastic parameters showed very good similarity in topology and geometry with the corresponding zones in the histological slices. Therefore, combined utilization of linear and nonlinear elastic parameters has confirmed the expectation of many authors that nonlinear elastic behavior is a very important characteristic of breast tissues that can provide useful information having a critical importance for a reliable clinical outcome.

In the general context of the development of elastographic techniques, in the recent review [42] presenting the development of elastography during the last three decades, there was a statement that, of high interest for elastography, is the “possibility that nonlinear parameters for normal tissues may differ from pathological cases, including cancers”. It was argued that differences in the structure and composition between tissues create a range of nonlinear parameters that can be used as a diagnostic tool [13,43,44,45]. In ultrasound elastography, the prospects of estimating nonlinear parameters attracted considerable attention in recent years [22,46,47,48,49,50,51,52,53,54,55,56,57,58]. Among promising directions of elastography development for the next decade, the abovementioned review [42] pointed out that “creating imaging strategies for estimating nonlinear parameters in a user-independent, accurate, ergonomic, and high resolution platform remains an important goal with many promising clinical applications”. In this regard, the OCE-approach proposed in a series of works [15,16,17] has already enabled an efficient and practically operable method for obtaining nonlinear stress–strain with a high spatial resolution. The above-presented results of OCE-based high-selectivity differentiation and automated segmentation of tumor tissue subtypes demonstrate that the combined usage of linear and nonlinear elastic parameters confers to OCE unprecedented diagnostic possibilities comparable to results of histological segmentation. However, in contrast to invasive, laborious, and time-consuming histology, the OCE examinations can be made in minutes, do not require special preparation of freshly excised samples or can even be feasible *in vivo,* at least in animal experiments with model tumors (like in [31]).

The next remark relates to compression elastographic techniques in general, including OCE. From the general viewpoint of materials science, most biological tissues relate to the class of poroelastic materials. They are strongly saturated with water with typical mass content ~70–90%, but mostly this water is in a bounded state with some free water localized in macroscopic gaps and channels. The closing of these pores/gaps under compression leads to an increase in the Young’s modulus (i.e., nonlinearity of stress–strain curves). By combining OCE-based strain and elasticity assessment, it becomes possible to extract information about microstructural changes in biological materials (e.g., corneal tissue and cartilage as demonstrated in [59,60]), using analogies from the characterization of crack-containing rocks. The squeezing of water from the gaps/pores, generally speaking, is a relaxation process with a broad spectrum of relaxation times. However, both the OCE method and compression USE utilize quasistatic compression (with characteristic frequencies ~ several Hz). Direct tests in OCE and longtime successful usage of USE demonstrate that for such compression rates, the observed strains are fairly well reproducible and lead to reproducible diagnostic conclusions. However, for higher characteristic frequencies (for oscillations in the range of hundreds Hz and kHz range), there are known special studies focused on estimation of relaxation times that can also be used for diagnostic applications [61].

Finally, it can be mentioned that the robust approach to OCT visualization of local strains based on the vector method proposed in [11,12] used here, in addition to the assessment of tissue elasticity in compression OCE, also opened a broad range of possibilities for studying various kinds of deformation processes in biological tissues, e.g., fairly slowly varying strains caused by drying [62], internal stresses [63], heating (e.g., by laser irradiation) [64], and osmotic effects [65].

## 5. Conclusions

In this study, we proposed a method for the quantification of the Young’s modulus of the tissue and its nonlinear parameters. The performed study showed that different morphological components of breast cancer tissue (i.e., fibrosis without cancer cells, fibrosis with small amount of cancer cells, zones with large agglomerates of cancer cells typical of IDC and ILC cancers, as well as zones of hyalinosis) demonstrate distinctly different combinations of the Young’s modulus and nonlinear elastic parameter. Their combined usage enables clear differentiation of these tissue subtypes; whereas taken separately, the Young’s modulus and the nonlinearity parameter are not sufficient to reliably distinguish them.

The obtained results indicate a high potential of the presented OCE technique for accurate intraoperatively feasible diagnosis of tumors and precise detection of a clean (“negative”) boundary for breast cancer resection during organ-conserving surgical operations. Certainly, in a more general sense, the developed technique can be used for a broad range of soft materials, first of all in further investigations of fundamental aspects of biomechanics including both linear and nonlinear elastic properties of various types of nontumoral and tumoral tissues.

## Data Availability

The data presented in this study are available on request from the corresponding author. The data are not publicly available due to proprietary rules.
